# Changes in the incidence and prevalence of systemic lupus erythematosus between 1990 and 2020: an observational study using the Clinical Practice Research Datalink (CPRD)

**DOI:** 10.1136/lupus-2024-001213

**Published:** 2024-07-27

**Authors:** Jessica Ellis, Neil McHugh, John D Pauling, Ian N Bruce, Rachel Charlton, Anita McGrogan, Sarah Skeoch

**Affiliations:** 1 Department of Life Sciences, University of Bath, Bath, UK; 2 Rheumatology, Royal National Hospital for Rheumatic Diseases, Royal United Hospitals Bath NHS Foundation Trust, Bath, UK; 3 Department of Rheumatology, North Bristol NHS Trust, Westbury on Trym, UK; 4 Centre for Musculoskeletal Research, The University of Manchester, Manchester, UK

**Keywords:** Epidemiology, Lupus Erythematosus, Systemic, Prevalence, Incidence

## Abstract

**Objective:**

To obtain updated estimates on the incidence and prevalence of systemic lupus erythematosus (SLE) in the UK, over the period 1990–2020, using the Clinical Practice Research Datalink (CPRD).

**Methods:**

This was a retrospective cohort study using the CPRD covering the period 1990–2020. A case ascertainment algorithm was developed in line with best practice recommendations for observational research. Incidence was calculated per 100 000 person-years and point prevalence (at the mid-year point) calculated per 100 000. Results were stratified by sex.

**Results:**

9443 SLE cases were identified. 5278 incident cases were identified (4538 women, 740 men). The overall incidence rate was 5.47 (95% CI 5.33 to 5.62) cases per 100 000 person-years. Incidence rates decreased slightly across the study period, which was more pronounced for women than men. Point prevalence increased over time, from 21.4 (95% CI 17.68 to 25.67) per 100 000 in 1990 to 107.14 (95% CI 103.26 to 111.12) per 100 000 in 2020.

**Conclusions:**

The observed fivefold increase in prevalence of SLE over the last 30 years, in the context of a modest decline in incidence rate, may suggest improved outcomes in SLE and has important implications for healthcare service delivery and planning in the UK.

**WHAT IS ALREADY KNOWN ON THIS TOPIC**

- Globally rises in prevalence of systemic lupus erythematosus (SLE) have been described alongside static or declining incidence rates. The last published updates on UK SLE epidemiology are outdated, with more contemporaneous estimates overdue.

**WHAT THIS STUDY ADDS**

- Incidence of SLE in the UK has shown a modest decline over the period 1991–2019.
- Prevalence of SLE in the UK has increased significantly over this same period.

**HOW THIS STUDY MIGHT AFFECT RESEARCH, PRACTICE OR POLICY**

- The observed increasing UK SLE prevalence over this period may reflect many factors, including enhanced detection of milder disease and/or improved survival outcomes.
- The demographics, phenotype and comorbidity burden of UK patients with SLE may, therefore, also be changing over time, necessitating changes to the management of these patients in practice.

## Introduction

 Systemic lupus erythematosus (SLE) is a chronic multisystem auto-immune rheumatic disease, with innumerate clinical manifestations, typically affecting women. SLE can have an unpredictable clinical course, typified by periods of remission interspersed with disease flares. Flares may affect almost any region of the body and can be life threatening. Morbidity of patients with SLE remains high, with implications for quality of life,^1^ employment prospects^2^ and family life.^3^ Consequently, patients living with SLE will often require lifelong input from healthcare services, with associated economic costs for patients and healthcare systems.^4^

A recent systematic review of the global epidemiology of SLE^5^ highlighted considerable variations in estimates of SLE incidence and prevalence. Even within Europe, reported incidence estimates have varied between 1.5 and 7.4 per 100 000 person-years, with point prevalence rates ranging between 29 and 210 per 100 000 patients. Differences in case definitions, methodological approach and data sources used may explain much of this variation. The only UK study referenced in this review^6^ used the Clinical Practice Research Datalink (CPRD), reporting an incidence of 4.9 per 100 000 patient years, and 2012 point prevalence of 97 per 100 000 patients. The authors observed a trend for decreasing incidence and increasing prevalence over time. These estimates were higher than those produced by previous studies^7 8^ in a previous iteration of the same database, potentially due to different study parameters and case definitions. Rees *et al*^6^ used data from 1999 to 2012 and as such, an updated estimate of current UK SLE epidemiology will be valuable for healthcare service delivery and future planning. Here, we present updated figures for longer term trends in the UK SLE incidence and prevalence between 1990 and 2020.

## Methods

### Clinical Practice Research Datalink

The CPRD provides health data for research from primary care practices across the UK.^9^ The dataset contains deidentified data, including medical diagnoses, prescriptions and test results, corresponding to nearly 20 million patients.^10^ CPRD allows for the assessment of longitudinal health trends, with a median follow-up time within CPRD GOLD (the CPRD dataset collected via Vision software in GP practices) of 13 years.^11^ The validity and representativeness of CPRD data for observational research are well established.^9^

### Study design and setting

The study was a retrospective cohort study performed within CPRD GOLD. The study period was from 1 January 1990 to 31 December 2020, using the dataset extracted in January 2021 build. The RECORD guidelines^12^ for studies conducted using observational routinely collected health data were followed. The study received institutional approval (University of Bath) and was approved under the CPRD Research Data Governance (RDG) process (protocol 21_000697).

### Study population

Participants were eligible for the study if they were permanently registered adults (age ≥18 at study entry) contributing data of a standard suitable for research with at least 12 months of continuous data within the study period.^9^ Index date was set as the earliest record of an SLE diagnostic Read code or code for immunomodulatory prescription (eg, methotrexate, hydroxychloroquine). Study entry was set as the index date. Study exit was set as the latest of death or their last data collection point (either the end of data collection or when they or their practice left the CPRD). Individual patient consent is not required for using the CPRD, but patients opting into the National Data Opt-out are not included.

### Case ascertainment algorithm

**Figure 1:**
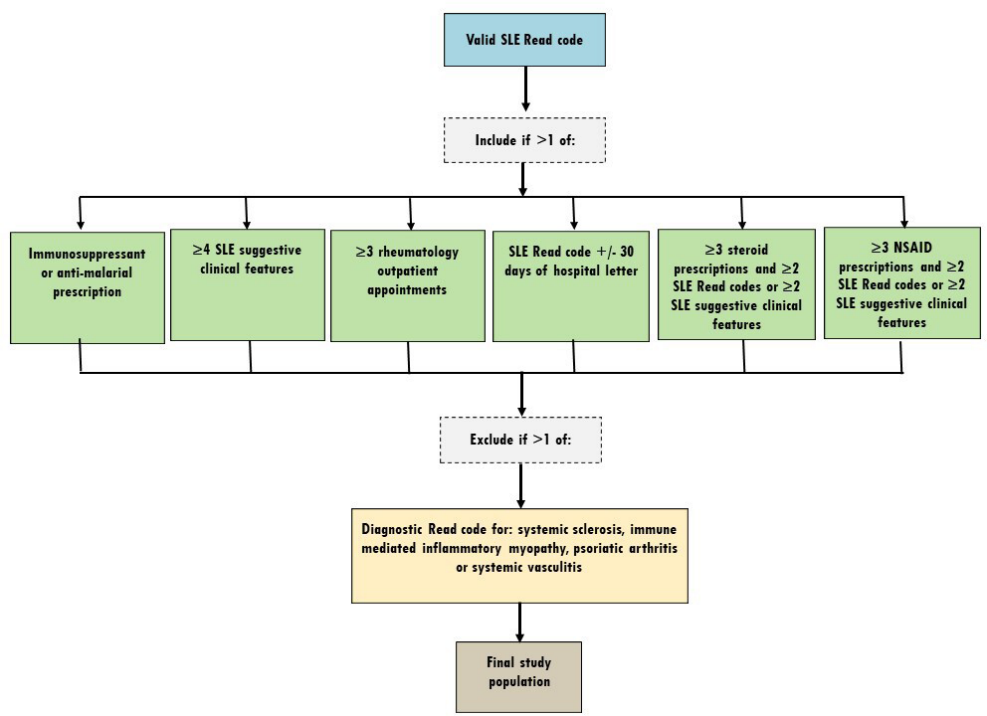
SLE case ascertainment algorithm. NSAID, non-steroidal anti-inflammatory drug.

An algorithm (see figure 1) was developed to optimise our ability to identify true SLE cases within the database. Various strategies for SLE case identification within the CPRD have been used. These encompass the presence of a single SLE Read code^613^,^15^ to algorithmic approaches requiring additional evidence for confirmation of an SLE diagnosis.^16^,^19^ Other studies of rheumatic diseases have highlighted that diagnostic Read codes alone may be insufficient to accurately identify true cases within the CPRD.^20^,^22^ Typically diagnostic Read codes are assigned to patient records by primary care clinical teams following confirmation of a diagnosis, that is, after review by a rheumatologist. However, coding errors can occur, emphasising the benefit of incorporating additional evidence for true case identification. To date, no studies reporting external validation of SLE cases have been reported. For this study, we adapted the algorithm presented by Nightingale *et al*^16^ in 2017, as outlined below.

#### SLE Read codes

Potential SLE Read codes were identified from the CPRD dictionary and from review of code lists reported in previous CPRD studies.^6 15 16^ Code lists were reviewed independently by two rheumatologists (JE and SS) and those with agreement retained. Codes relating to limited cutaneous lupus, tuberculoid lupus and lupus anticoagulant were excluded. Codes for subacute cutaneous lupus were kept; in our experience, many patients with systemic lupus will have this manifestation, compared with more specific cutaneous involvement such as discoid lupus.

#### Confirmatory evidence

We searched for clinical and immunological features suggestive of SLE, for example, pericarditis diagnostic codes, anti-nuclear antibody codes) based on the 2019 EULAR-ACR classification criteria.^23^ Immunosuppressants were amended to include SLE-related medications, which are now more widely used (addition of Leflunomide, Mycophenolate Mofetil, Rituximab and Tacrolimus and Belimumab). In recognition of recent efforts to minimise cumulative glucocorticoid exposure in the management of SLE, we removed evidence of ≥3 months continuous oral glucocorticoids but incorporated repeat glucocorticoid or non-steroidal anti-inflammatory prescriptions combined with multiple SLE Read codes, or multiple EULAR-ACR criteria. The entire patient record was reviewed for confirmatory evidence. Test data (eg, white cell count) had inclusion thresholds set based on unit types and expected normal values. The full algorithm used is shown in figure 1.

The list of Read codes for SLE and confirmatory evidence, used in this study, are available via the University of Bath Research Data Archive (*https://researchdata.bath.ac.uk/*) or by contacting the corresponding author.

#### Exclusion criteria

Patients were excluded if they had a record of a non-SLE rheumatic diagnosis after their SLE Read code. These diagnoses included: systemic sclerosis, immune-mediated inflammatory myopathy, psoriatic arthritis and systemic vasculitis (including Behçet’s disease). Rheumatoid arthritis, Sjögren’s syndrome and antiphospholipid syndrome were not excluded due to frequent co-existence of these diseases with SLE.

### Co-variates

Records were reviewed for weight, BMI and smoking status, with the closest result to index date taken. Smoking status was categorised as smoker, never-smoker or ex-smoker using an in-house algorithm. Records were reviewed for relevant ethnicity Read codes then categorised (White, Black, South Asian, Other). Patients with conflicting code categories or missing data were labelled as unknown.

### Outcome measurement

Patients with at least 12 months of data preceding index date with no SLE Read code were eligible to be incident cases. This 12-month data period is in keeping with other studies of incident SLE populations within the CPRD.^617^,^19 24^ Incidence of SLE was calculated by year of diagnosis (1991–2019) and by age band at diagnosis, using the rest of the valid (defined as permanently registered patients, ≥18 at study entry, at least 1 year of up to standard data during the study period) population present in the CPRD. Incidence is reported per 100 000 person-years. Stratified incidence rate estimates were obtained by age and sex. Prevalent cases included those joining the study with an existing SLE diagnosis, or those initially diagnosed as incident who in subsequent years became prevalent. Point prevalence rates at the mid-year point (1 July) were evaluated using the mid-year SLE and total population counts as the numerator and denominator, respectively.

### Statistical analysis

Incidence and prevalence rates (by age, sex, year) with 95% CIs were calculated using the epiR package.^25^ Method was set as exact. All data management and analysis were performed using R statistical software (v4.0.3; R Core Team 2020).

### Patient and public involvement

Patients and the public were not involved in the design or conduct of this study nor reporting or dissemination of the results.

## Results

### SLE cohort

A study population of 11 409 684 individuals was identified in CPRD GOLD. This contained 12 376 patients with a valid SLE diagnostic code. Confirmatory evidence was present for 9648 patients. A prescription for an SLE-specific medication was the most frequent piece of supplementary evidence (n=6582). The least frequent piece of supplementary evidence was the presence of ≥4 EULAR-ACR 2019 classification criteria (n=1529). The majority of cases had ≥1 piece of supplementary evidence recorded. A summary of counts per type of confirmatory evidence is presented in online supplemental table S1. Following exclusions (n=205), the final cohort contained 9443 cases (see online supplemental figure S1 for flow diagram summary of patients included in the study).

### Cohort demographics

Characteristics of the final SLE patient cohort are presented in online supplemental table S2. As expected, SLE was more common in women than men (8258 vs 1158 cases). Women were diagnosed at a younger age than men (median 43 vs 50 years). Male cases were more likely to be a current or previous smoker than women (63.64% vs 47.98%). There was a significant amount of missing data for BMI and ethnicity (54.4% and 52.7%, respectively). Where recorded, the most common ethnic category was white. The percentage of patients with an ethnicity category recorded as an ethnic minority was only 6.3%. Mean and median age (at the mid-year point, 1 July) of the current SLE cohort at 5-year intervals across the study period are presented in online supplemental table S3. The average age of the SLE population increased across the study period (1990 mean age=45.0, median age=44. 2000 mean age=57.9, median age=58).

Incidence rates (per 100 000 person-years) and point prevalence (per 100 000) are presented in table 1, alongside absolute counts and 95% CIs for estimates.

**Table 1:** Incidence and prevalence of SLE by year 1990–2020

Year	Number incident SLE cases	Person-years	Incidence rate per 100 000 person-years (95% CI)	Number prevalent SLE cases	Point prevalence per 100 000 (95% CI)
1990	–	–	–	116	21.4 (17.68 to 25.67)
1991	39	753 679	5.17 (3.68 to 7.07)	191	25.24 (21.79 to 29.09)
1992	46	892 119	5.16 (3.78 to 6.88)	273	31.23 (27.64 to 35.17)
1993	46	101 918	4.51 (3.3 to 6.02)	350	34.32 (30.82 to 38.11)
1994	57	109 331	5.21 (3.95 to 6.75)	428	39.53 (35.88 to 43.46)
1995	53	117 166	4.52 (3.39 to 5.92)	515	44.09 (40.36 to 48.07)
1996	72	132 422	5.44 (4.25 to 6.85)	633	47.44 (43.81 to 51.28)
1997	87	156 765	5.55 (4.45 to 6.85)	788	51 (47.5 to 54.69)
1998	111	181 522	6.11 (5.03 to 7.36)	962	53.85 (50.51 to 57.37)
1999	124	222 178	5.58 (4.64 to 6.65)	1215	55.75 (52.66 to 58.97)
2000	177	277 007	6.39 (5.48 to 7.4)	1575	57 (54.22 to 59.89)
2001	206	3 175 621	6.49 (5.63 to 7.44)	1924	61.14 (58.44 to 63.94)
2002	258	370 777	6.96 (6.14 to 7.86)	2366	64.16 (61.6 to 66.8)
2003	255	420 606	6.06 (5.34 to 6.85)	2810	67.24 (64.78 to 69.78)
2004	252	468 865	5.37 (4.73 to 6.08)	3230	68.65 (66.3 to 71.05)
2005	279	486 354	5.74 (5.08 to 6.45)	3494	71.67 (69.31 to 74.09)
2006	267	495 055	5.39 (4.77 to 6.08)	3696	74.98 (72.58 to 77.43)
2007	305	503 333	6.06 (5.4 to 6.78)	3976	79.26 (76.82 to 81.76)
2008	294	506 977	5.8 (5.16 to 6.5)	4241	83.63 (81.14 to 86.19)
2009	238	509 782	4.67 (4.09 to 5.3)	4435	86.84 (84.3 to 89.43)
2010	287	506 876	5.66 (5.03 to 6.36)	4558	89.67 (87.08 to 92.31)
2011	318	499 833	6.36 (5.68 to 7.1)	4692	93.44 (90.79 to 96.15)
2012	264	496 640	5.32 (4.69 to 6)	4815	96.64 (93.93 to 99.4)
2013	264	479 323	5.51 (4.86 to 6.21)	4788	99.53 (96.74 to 102.39)
2014	202	449 770	4.49 (3.89 to 5.16)	4522	100.24 (97.34 to 103.21)
2015	198	403 842	4.9 (4.24 to 5.64)	4151	101.86 (98.79 to 105.01)
2016	166	348 648	4.76 (4.06 to 5.54)	3617	103.57 (100.22 to 107)
2017	160	319 613	5.01 (4.26 to 5.84)	3315	104 (100.49 to 107.6)
2018	134	304 508	4.4 (3.69 to 5.21)	3226	106.33 (102.7 to 110.06)
2019	119	294 198	4.04 (3.35 to 4.84)	3154	106.59 (102.91 to 110.38)
2020	–	–	–	2878	107.14 (103.26 to 111.12)

### Incidence

5278 incident cases were identified (4538 women and 740 men). The overall incidence rate was 5.47 (95% CI 5.33 to 5.62) cases per 100 000 person-years. Incidence slightly increased between 1991 and 2002, where a peak incidence rate of 6.96 (95% CI 6.14 to 7.86) per 100 000 person-years was seen. A general trend for decreasing incidence was then observed, with the lowest incidence of 4.04 (95% CI 3.35 to 4.84) per 100 000 person-years in 2019. This was largely influenced by women, with male incidence rates showing more stability over the study period.

**Figure 2:**
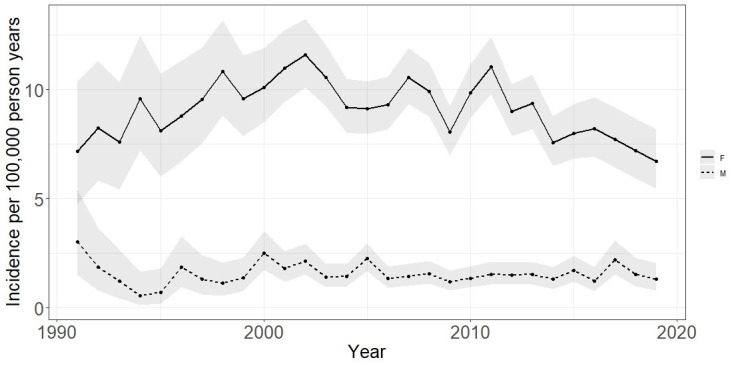
Incidence rate of SLE between 1991 and 2019 stratified by sex. Grey shading indicates 95% CIs for estimates.

**Figure 3:**
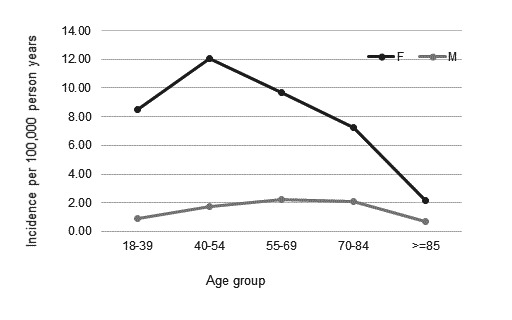
Incidence per 100 000 person-years by age group and sex.

Incidence rates were higher for women than men across the whole study period (see figure 2). The lowest incidence rate for males was 0.57 (95% CI 0.12 to 1.66) per 100 000 person-years in 1994, compared with the lowest for women of 6.72 (95% CI 5.47 to 8.17) per 100 000 person-years in 2019. The highest incidence rate for males was 3.03 (95% CI 1.51 to 5.42) per 100 000 person-years seen in 1991, compared with the highest for women of 11.59 (95% CI 10.11 to 13.23) per 100 000 person-years in 2002 (see online supplemental table S4).The highest incidence rate by age group across the whole cohort was seen in the 40–54 age group (see figure 3). For women, the 40–54 age group had the highest incidence rate, of 12.08 per 100 000 person-years. For men, the 55–69 age group had the highest incidence rate of 2.22 per 100 000 person-years.

The greatest difference between sexes regarding age group at incident diagnosis was seen in the 18–39 age group, with a women to men ratio of over 9. This reduced with increasing age, reflecting the trend for older male age at diagnosis. For both sexes, diagnosis over the age of 85 was infrequent.

### Prevalence

**Figure 4:**
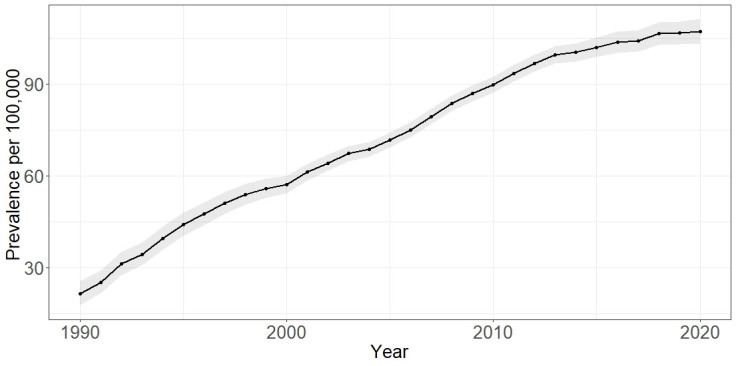
Prevalence (cases per 100 000) of SLE in the CPRD 1990–2020. Grey shading indicates 95% CIs for estimates. CPRD, Clinical Practice Research Datalink.

9443 prevalent cases were identified (8285 women, 1158 men). Point prevalence increased over time, from 21.4 (95% CI 17.68 to 25.67) per 100 000 in 1990 to 107.14 (95% CI 103.26 to 111.12) per 100 000 in 2020 (see figure 4).

**Figure 5:**
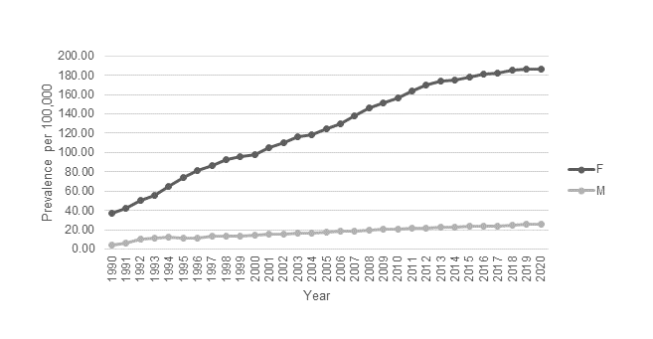
Prevalence (cases per 100,000) of SLE in the CPRD 1990–2020 stratified by sex. CPRD, Clinical Practice Research Datalink.

Prevalence increased for both women and men during the study period (see figure 5). Women saw an increase from 37.13 (95% CI 30.34 to 44.99) per 100 000 in 1990 to 186.67 (95% CI 179.48 to 194.07) per 100 000 in 2020. Men saw an increase from 4.58 (95% CI 2.37 to 8.00) per 100 000 in 1990 to 25.69 (95% CI 23.04 to 28.57) per 100 000 in 2020.

There were differences observed when prevalence was stratified by age and sex (see online supplemental figure S2). The highest prevalence by age group for women was seen in the 60–64 age group (224.63 per 100 000 (95% CI 215.24 to 234.33)). The prevalence in the male population was low and prevalence was similar between the ages of 50 and 80 years old.

## Discussion

Between 1991 and 2019, we observed an overall incidence rate of 5.47 cases per 100 000 patient-years. The most common age group for incident SLE was 40–54. There was a higher incidence and prevalence of SLE in women than men. UK prevalence of SLE, for men and women, has increased over time.

We observed a higher overall incidence rate than that of Rees *et al* in the last CPRD study reporting trends in UK SLE epidemiology.^6^ Methodological differences between studies may account for this. We used a more comprehensive list of SLE Read codes (addition of F396100 and M154700 Read codes), which may have increased the number of individuals eligible for classification as SLE cases. We believe our case ascertainment strategy will have mitigated the risk of case misclassification arising from a more comprehensive diagnostic code list. Our observed incidence rate lies within the ranges recently identified for SLE globally,^5^ lending further weight to our estimates.

We observed a trend for decreasing incidence over time, although less pronounced than that of Rees *et al*. Different durations of study periods (1991–2019 vs 1999–2012) may account for this, with our longer duration capturing fluctuations in incidence over time. Globally discordant observations of SLE incidence trends exist, with decreases,^26^ stability^27^ and increases^28^ described. Direct comparison between these observations is limited due to methodological, population and healthcare differences between studies. The decline in incidence observed in the current study may merely represent a longer term fluctuating pattern, which a further period of follow-up will determine. However, if the observed decline is genuine, one hypothesis could be that improvements in diagnostics and wider recognition of other connective tissue diseases have led to less patients being misclassified as having SLE, in more recent years. Further analysis over time in this CPRD cohort and others will be required to further investigate whether incidence is truly declining.

Incident SLE was higher in women than men. This was seen across all time points and age groups at diagnosis and confirms the known predilection of SLE for women.^29^ The mean age at diagnosis was 44.72 for women and 49.66 for men. The most common overall age group for incident cases was 40–54, in keeping with estimates from several other European registry-based studies.^30^,^32^ This is older than has been described in several large international^33^ and UK^34 35^ SLE cohort studies. Cohort studies, particularly from secondary care, may include more severe disease, which is associated with younger age at diagnosis.^36^ These differences may, therefore, reflect population dissimilarities in these contrasting settings. Still, this may be reflective of a better awareness of SLEs ability to present at older ages, accompanied by developments in diagnostic testing and classification criteria that better support diagnosis in less ‘typical’ patient populations. Alternatively, the older age observed in the CPRD could reflect delay between clinical diagnosis and diagnostic code recording in primary care. However, it seems unlikely that this delay would account for more than a few years, and, therefore, not adequately explain these differences.

Men were more likely to be older at diagnosis, with peak incidence seen in the 55–69 age group. Other registry-based studies have observed a similar trend for male older age at diagnosis.^6 37^ This may reflect differences in SLE pathophysiology between sexes. Alternatively, given SLE is known to be more common in women, men could be more likely to experience diagnostic delay due to under-recognition by physicians. However, several large studies have actually reported shorter delay to diagnosis for men,^38^,^40^ suggesting true sex differences may exist.

Male incidence rates showed less variation between age groups than women, a pattern which has been observed in other settings.^37 41^ The greatest difference between sexes regarding age group at incident diagnosis was seen in the 18–39 age group, with the female to male ratio 9.4. This reduced with increasing age, reflecting the trend for older male age at diagnosis. For both sexes, diagnoses over the age of 85 were infrequent.

Our study shows the prevalence of SLE in the UK has increased over time. Our estimates of prevalence are slightly higher than that of Rees *et al*,^6^ most likely reflecting our longer study duration,^42^ but display a similar trend to suggest an increase over time. This has been observed in other settings,^26 43 44^ suggesting it to be a true phenomenon. Earlier diagnosis, better treatments and improved survival may all account for the increased incidence. We observed an increase in the average age of the SLE cohort across the study period, which again may be suggestive of improving survival. Changing trends in ethnic composition of the UK population have been observed, with a rise in ethnic minority populations, known to have higher rates of SLE.^45^ These changes may be contributing to increased prevalence over time. However, we were unable to evaluate this due to the degree of missing data on ethnicity within CPRD. Irrespective of the cause, the observed increase in prevalence is an important observation for future UK health economic planning.

There are some key strengths and limitations of this study. Strengths include a long study period, allowing comprehensive observation of trends in UK SLE epidemiology over nearly a 30-year period. It should be noted, however, that the CRPD population size was significantly smaller in the earliest phase of the study (~750 000 patients in 1990 vs >1 000 000 after 1995). Thus, estimates of incidence and prevalence may be less accurate in this time window, than later in the study. This should be borne in mind when interpreting results.

Our comprehensive case ascertainment algorithm follows good practice pertaining to the identification of cases using routinely collected health data.^12^ The CPRD as a data source may allow inclusion of milder cases of SLE that may be excluded from hospital-based cohorts, giving a more accurate assessment of SLE epidemiological trends across the entire UK population. We did not perform external validation of cases in this study, due to cost and limitations on primary care capacity for case validation for the CPRD during the COVID-19 pandemic. Therefore, it remains a possibility that we have incorrectly classified non-SLE diagnoses as cases. We believe our case ascertainment strategy has mitigated this risk but will not have removed it completely. To date, there are no published externally validated methods for identifying true SLE cases within the CPRD, which may be a helpful area of future study to strengthen work in this area.

A key limitation of this study is the large amount of missing ethnicity data. We did not use linked Hospital Episode Statistics data in this study, and as previously discussed, ethnicity data available within CPRD alone were poor. This restricts our ability to evaluate the influence of ethnicity on trends in prevalence and incidence. Finally, given this is an observational study, no causality can be attributed to the trends observed. It remains possible that trends seen reflect differences in primary care coding practices over time rather than true patterns in UK SLE epidemiology. However, coding changes would not fully explain the differential trends observed for incidence and prevalence. New initiatives such as the inclusion of new connective tissue diseases in the British Society for Rheumatology National Early Inflammatory Arthritis Audit,^46^ alongside other health registry platforms such as OpenSafely,^47^ will provide complementary sources of data to the CPRD for interrogating trends in UK SLE epidemiology and are, therefore, important settings for future research to be conducted in.

## Conclusion

Utilising primary care records from the UK population spanning a 30-year period, we have explored the changing epidemiology of SLE. We observed a large increase in prevalence of SLE alongside a modest decline in incidence rate. This may indicate improving outcomes for UK SLE patients as well as implications for healthcare service delivery and planning in the UK. Further study into the impact of ethnicity on these patterns is important to better understand the requirements of the current UK SLE population.

## supplementary material

10.1136/lupus-2024-001213 online supplemental file 1

## Data Availability

Data are available upon reasonable request.
