# Supplementary material for: Changes in the incidence and prevalence of systemic lupus erythematosus between 1990 and 2020: an observational study using the Clinical Practice Research Datalink (CPRD)
Source: Lupus Sci Med. 2024 Jul 27;11(2):e001213. doi: 10.1136/lupus-2024-001213 (PMC11284910; doi:10.1136/lupus-2024-001213)

Supplementary Evidence File

Supplementary Table S1: Counts per type of confirmatory evidence

	All		
	Males	Females	Total
>=1 prescription for DMARD	670	5912	6582
>=4 ACR criteria	152	1377	1529
=2/3 ACR criteria	500	3774	4274
SLE diagnosis within (+/-) 30 days as OP hospital letter/appt	605	3682	4287
SLE diagnosis within (+/-) 30 days as IP stay/ discharge summary	64	357	421
SLE diagnosis within (+/-) 30 days as hospital letter and or discharge summary	669	4039	4708
>=3 NSAID prescription	825	5877	6702
>=3 Steroid prescription	676	4704	5380
=2/3 ACR + >=3 NSAID	272	2346	2618
=2/3 ACR + >=3 Steroid	221	1811	2032
=>2 SLE records + >=3 NSAID	297	2704	3001
=>2 SLE records + >=3 Steroid	318	2619	2937
>=3 Rheumatology OPD post diagnosis	384	3993	4377

Supplementary Table 2 S2: Characteristics of the final SLE cohort

	Males (n)	Males (%)	Females (n)	Females (%)	Total (n)
Age at diagnosis (mean)	49.66		44.72		
Age at diagnosis (median, range)	50 (18-91)		43 (18-109)		
18-39	336	29.02	3434	41.45	3770
40-54	366	31.61	2663	32.14	3029
55-69	321	27.72	1525	18.41	1846
70-84	129	11.14	618	7.46	747
85+	6	0.52	45	0.54	51
Total (n)	1158	[redacted]	8285	[redacted]	9443
Ethnicity					
WHITE	439	37.91	3434	41.45	3873
BLACK	15	1.30	189	2.28	204
S. ASIAN	27	2.33	262	3.16	289
OTHER	8	0.69	93	1.12	101
Unknown	669	57.77	4307	51.99	4976
BMI					
<20	35	3.02	370	4.47	405
20-24	144	12.44	1323	15.97	1467
25-29	220	19.00	1098	13.25	1318
30-34	93	8.03	551	6.65	644
35-39	32	2.76	239	2.88	271
≥40	12	1.04	185	2.23	197
Unknown	622	53.71	4519	54.54	5141
Smoking status					
CURRENT	420	36.27	2406	29.04	2826
EX	317	27.37	1569	18.94	1886
NEVER	408	35.23	4220	50.94	4628
Unknown	13	1.12	90	1.09	103

Supplementary Table 3 S3: Average age of the SLE cohort by 5-year interval across the study period

TIME POINT	NUMBER OF SLE CASES	MEAN AGE	MEDIAN AGE
01-Jul-90	993	45.0	44
01-Jul-95	1,934	47.2	47
01-Jul-00	3,195	49.5	49
01-Jul-05	4,588	51.9	51
01-Jul-10	5,377	53.7	53
01-Jul-15	4,506	55.6	55
01-Jul-20	2,878	57.9	58

Average ages for the current eligible SLE population were taken at the mid- year point per every 5 years of the study period. Eligible is defined as being a current case at the mid-year point (i.e. after index date and before rcens. rcens is a CPRD supplied metric that is usually the earliest of date of death or the date the patient or practice leaves the CPRD.

Supplementary Table 4 S4: Incidence per 100,000 patient years by sex and age group

	Female						Male						Total
**Year**	**18-39**	**40-54**	**55-69**	**70-84**	**>=85**	**Total**	**18-39**	**40-54**	**55-69**	**70-84**	**>=85**	**Total**	
1991	6.82	9.52	9.16	3.35	0.00	7.17	1.35	2.05	6.91	5.01	0.00	3.03	**5.17**
1992	8.71	8.01	8.95	8.45	0.00	8.24	0.57	3.44	3.51	0.00	0.00	1.86	**5.16**
1993	6.62	13.17	8.89	1.24	0.00	7.60	0.50	2.25	0.00	3.63	0.00	1.22	**4.51**
1994	8.59	12.19	13.91	4.64	0.00	9.57	0.47	0.69	0.96	0.00	0.00	0.57	**5.21**
1995	7.66	11.26	9.56	3.24	4.32	8.12	0.44	0.64	0.89	1.56	0.00	0.70	**4.52**
1996	5.26	16.70	9.22	5.74	0.00	8.80	1.58	1.67	1.59	4.09	0.00	1.87	**5.44**
1997	7.92	14.49	11.66	4.94	0.00	9.55	1.67	0.93	1.33	1.16	0.00	1.31	**5.55**
1998	11.03	15.90	10.05	5.73	0.00	10.85	0.87	0.81	2.27	1.00	0.00	1.13	**6.11**
1999	8.04	14.01	10.41	7.18	0.00	9.58	0.71	0.99	3.21	1.65	0.00	1.38	**5.58**
2000	9.79	12.81	10.55	8.26	0.00	10.10	1.52	3.41	2.93	3.30	0.00	2.51	**6.39**
2001	9.46	14.84	12.13	8.12	4.57	10.98	1.00	1.84	2.19	4.02	0.00	1.80	**6.49**
2002	11.12	15.92	12.45	6.25	2.71	11.59	1.29	3.17	2.61	1.96	0.00	2.14	**6.96**
2003	10.70	13.07	11.25	7.53	0.00	10.57	0.89	2.26	0.90	1.30	6.11	1.40	**6.06**
2004	7.44	10.96	12.55	7.41	1.13	9.20	0.81	1.24	2.81	1.55	0.00	1.43	**5.37**
2005	8.52	11.72	8.98	7.87	2.08	9.12	1.35	2.21	3.46	3.39	0.00	2.25	**5.74**
2006	7.85	13.41	10.10	6.37	1.00	9.32	0.56	1.58	2.07	2.21	0.00	1.35	**5.39**
2007	10.96	12.66	10.30	8.92	0.00	10.55	1.32	1.41	1.29	2.17	2.19	1.45	**6.06**
2008	10.13	12.96	9.29	7.18	0.95	9.93	0.66	2.65	1.83	1.43	0.00	1.56	**5.80**
2009	7.49	9.53	9.77	5.46	2.83	8.06	0.33	1.10	2.55	1.77	0.00	1.19	**4.67**
2010	7.21	15.17	8.90	10.19	0.95	9.86	1.11	0.97	2.38	1.42	0.00	1.36	**5.66**
2011	10.37	13.57	11.51	8.91	4.75	11.06	0.57	1.97	2.03	2.88	0.00	1.54	**6.36**
2012	7.14	11.92	10.46	6.85	4.79	9.01	0.92	1.56	2.41	1.43	1.93	1.51	**5.32**
2013	10.70	11.01	7.37	7.33	4.96	9.37	0.96	2.07	1.15	2.91	0.00	1.53	**5.51**
2014	5.35	10.99	8.03	7.68	1.05	7.58	0.77	0.95	2.03	2.64	0.00	1.31	**4.49**
2015	7.74	8.48	9.24	6.75	4.68	8.00	0.72	2.15	2.89	1.67	0.00	1.71	**4.90**
2016	7.24	11.45	6.63	8.54	2.75	8.20	0.66	1.89	1.03	1.90	0.00	1.22	**4.76**
2017	9.10	6.68	7.28	7.88	4.63	7.72	1.44	2.54	3.09	2.03	2.78	2.22	**5.01**
2018	6.14	9.43	5.23	9.88	3.27	7.20	0.76	0.49	3.80	2.07	0.00	1.53	**4.40**
2019	5.59	8.57	8.63	4.58	1.68	6.72	0.59	1.55	1.79	1.57	2.91	1.31	**4.04**
**Total**	**8.50**	**12.08**	**9.66**	**7.27**	**2.19**	**9.26**	**0.90**	**1.75**	**2.22**	**2.11**	**0.66**	**1.56**	5.47

Supplementary Figure 1 S1: Flow diagram of patients included in the study

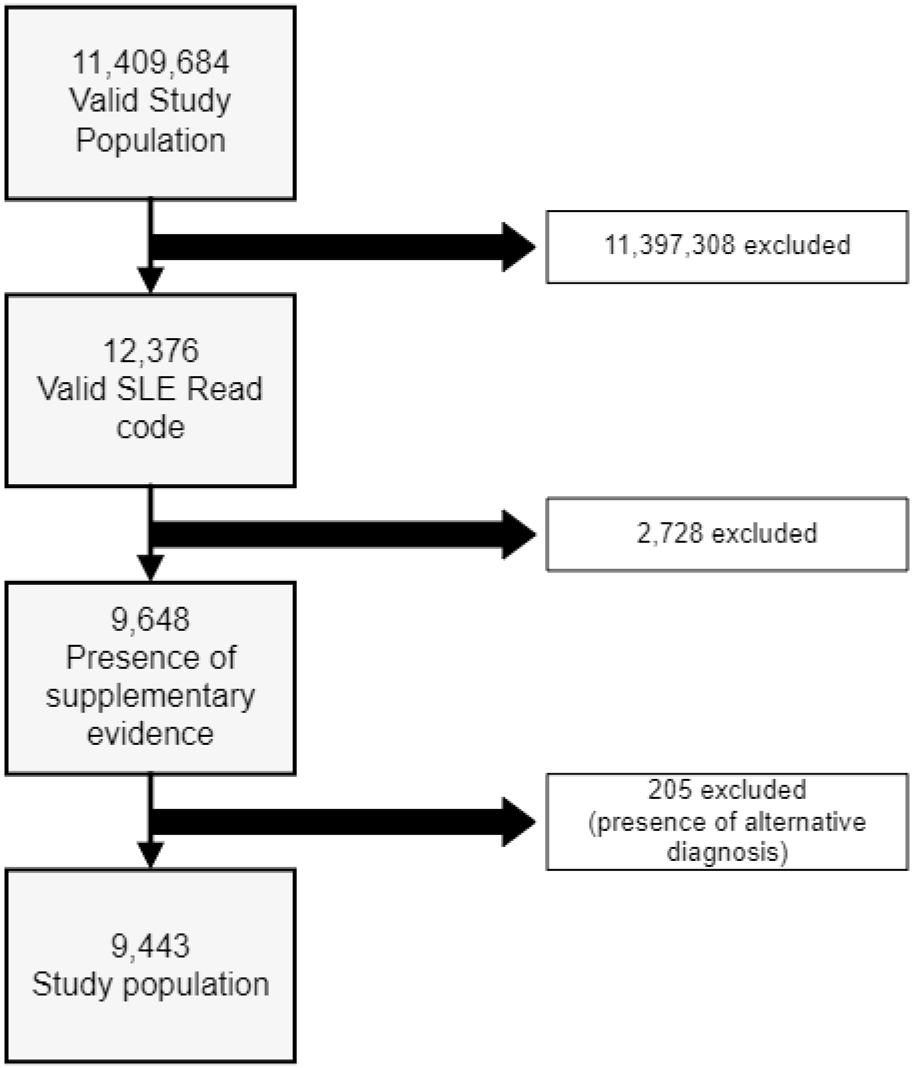

Supplementary Figure 2 S2: Prevalence per 100,000 stratified by age group

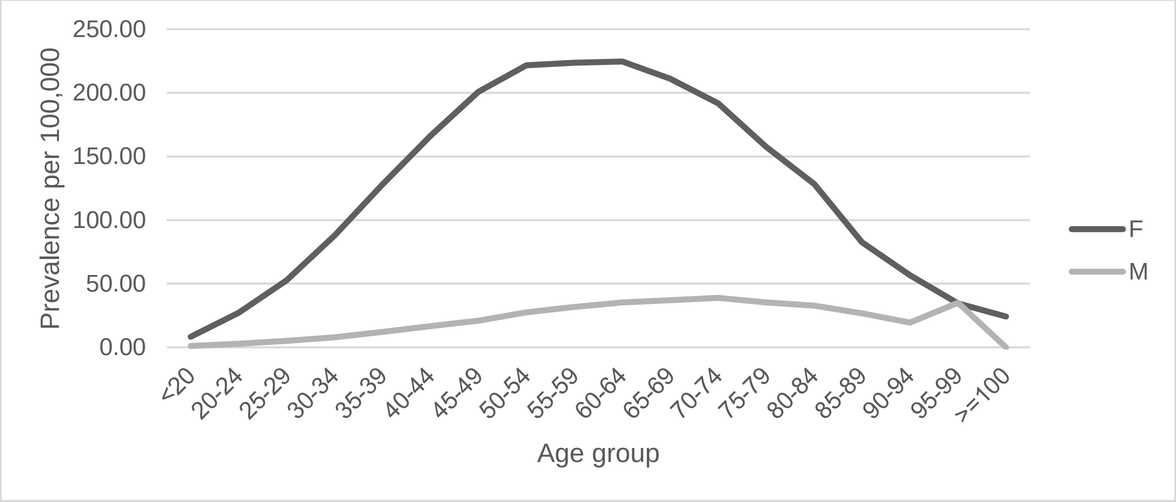

Supplement: online supplemental file 1 [file lupus-11-2-s001.pdf]
